# P02.76. Impedance plethysmographic (IPG) pulse signal analysis based personalized diagnostics & treatment simulating Ayurvedic pulse examination

**DOI:** 10.1186/1472-6882-12-S1-P132

**Published:** 2012-06-12

**Authors:** M Dhurandhar

**Affiliations:** 1 Centre for Development of Advanced Computing, Pune, India

## Purpose

Ayurveda advocates a holistic, individualized approach for disease diagnostics/treatment. Taking cognizance of physiological variabilities, Ayurveda devised a subjective method of pulse examination for health index assessment. As per Ayurveda, biological humors 'TriDosha' are fundamental psychosomatic principles responsible for health/diseases (Vata: Body Movements; Pitta: Heat, Metabolism; Kapha: Body Resistance). Doshas in homeostasis lead to health and imbalance lead to disease. Ayurvedic Pulse characteristics like Strength/Rate/Rhythm/Movement contribute to assessment of prodromal symptoms predicting diseases, diagnosing metabolic status, identifying causative agents, prognosis and lifespan. Integrative medical informatics tools are developed for IPG-based pulse signal analysis harnessing Ayurveda's clinical phenotyping principles and mathematical modeling with information technology for clinical assessment of pulse qualities.

## Methods

Pulse examination by Ayurveda doctor and pulse signal captured with IPG-based medical instrument. Correlation found between Power Spectral Density (PSD) of Heart Rate Variability (HRV) and dominant Dosha; pulse morphology patterns and parameters like strength, rate, rhythm, movement. This was done in a population of 1000 healthy patients.

## Results

Frequency bands of PSD of HRV showed signal intensity corresponding to Dosha levels (LF:Pitta; MF:Kapha; HF:Vata). Dosha corresponding to the band with maximum signal intensity found dominant (>85% accuracy). Ratios derived from morphology patterns' deflection points yielded characteristics like strength with high (>91%) accuracy.

## Conclusion

Few attempts are made to objectively evaluate autonomous nervous system functioning for patient benefit. With today's high stress-induced lifestyle, a major technological shift is emerging towards Integrative Medicine. Pharmacogenomics being in use to a limited degree, such multidisciplinary research can lead to evidence-based results, impacting optimal promotive/preventive/curative healthcare management by early changes in lifestyle, avoiding or lessening severity of diseases, and decreasing cost and risk of clinical trials by targeting persons capable of responding to a drug. Decreases in adverse drug reactions, failed drug trials, length of medication time, number. of medications taken for finding an effective therapy, and early detection will promote decrease in healthcare costs.

